# A Gambling Just-In-Time Adaptive Intervention (GamblingLess: In-The-Moment): Protocol for a Microrandomized Trial

**DOI:** 10.2196/38958

**Published:** 2022-08-23

**Authors:** Nicki A Dowling, Stephanie S Merkouris, George J Youssef, Dan I Lubman, Kathleen L Bagot, Chloe O Hawker, Hannah J Portogallo, Anna C Thomas, Simone N Rodda

**Affiliations:** 1 School of Psychology Deakin University Geelong Australia; 2 Melbourne Graduate School of Education University of Melbourne Melbourne Australia; 3 Turning Point and Monash Addiction Research Centre, Eastern Health Clinical School Monash University Melbourne Australia; 4 Data Drawer Consulting Sandringham Australia; 5 Psychology and Neuroscience Auckland University of Technology Auckland New Zealand; 6 School of Population Health University of Auckland Grafton New Zealand

**Keywords:** mobile health, mHealth, just-in-time adaptive intervention, ecological momentary intervention, microrandomized trial, gambling, addiction, treatment, intervention, protocol, relapse, mobile phone

## Abstract

**Background:**

The presence of discrete but fluctuating precipitants, in combination with the dynamic nature of gambling episodes, calls for the development of tailored interventions delivered in real time, such as just-in-time adaptive interventions (JITAIs). JITAIs leverage mobile and wireless technologies to address dynamically changing individual needs by providing the type and amount of support required at the right time and only when needed. They have the added benefit of reaching underserved populations by providing accessible, convenient, and low-burden support. Despite these benefits, few JITAIs targeting gambling behavior are available.

**Objective:**

This study aims to redress this gap in service provision by developing and evaluating a theoretically informed and evidence-based JITAI for people who want to reduce their gambling. Delivered via a smartphone app, *GamblingLess: In-The-Moment* provides tailored cognitive-behavioral and third-wave interventions targeting cognitive processes explicated by the relapse prevention model (cravings, self-efficacy, and positive outcome expectancies). It aims to reduce gambling symptom severity (*distal outcome*) through short-term reductions in the likelihood of gambling episodes (*primary proximal outcome*) by improving craving intensity, self-efficacy, or expectancies (*secondary proximal outcomes*). The primary aim is to explore the degree to which the delivery of a tailored intervention at a time of cognitive vulnerability reduces the probability of a subsequent gambling episode.

**Methods:**

*GamblingLess: In-The-Moment* interventions are delivered to gamblers who are in a state of receptivity (available for treatment) and report a state of cognitive vulnerability via ecological momentary assessments 3 times a day. The JITAI will tailor the type, timing, and amount of support for individual needs. Using a microrandomized trial, a form of sequential factorial design, each eligible participant will be randomized to a tailored intervention condition or no intervention control condition at each ecological momentary assessment across a 28-day period. The microrandomized trial will be supplemented by a 6-month within-group follow-up evaluation to explore long-term effects on primary (gambling symptom severity) and secondary (gambling behavior, craving severity, self-efficacy, and expectancies) outcomes and an acceptability evaluation via postintervention surveys, app use and engagement indices, and semistructured interviews. In all, 200 participants will be recruited from Australia and New Zealand.

**Results:**

The project was funded in June 2019, with approval from the Deakin University Human Research Ethics Committee (2020-304). Stakeholder user testing revealed high acceptability scores. The trial began on March 29, 2022, and 84 participants have been recruited (as of June 24, 2022). Results are expected to be published mid-2024.

**Conclusions:**

*GamblingLess: In-The-Moment* forms part of a suite of theoretically informed and evidence-based web-based and mobile gambling interventions. This trial will provide important empirical data that can be used to facilitate the JITAI’s optimization to make it a more effective, efficient, and scalable tailored intervention.

**Trial Registration:**

Australian New Zealand Clinical Trials Registry (ANZCTR) ACTRN12622000490774; https://www.anzctr.org.au/Trial/Registration/TrialReview.aspx?id=380757&isClinicalTrial=False

**International Registered Report Identifier (IRRID):**

PRR1-10.2196/38958

## Introduction

### Background

Gambling disorder (formerly pathological gambling) has been reclassified in the Diagnostic and Statistical Manual of Mental Disorders (Fifth Edition) as an addiction and related disorder alongside alcohol and substance use disorders [1]. Consistent with public health frameworks that conceptualize gambling problems across a continuum of risk [2], many jurisdictions, including Australia and New Zealand, use the term problem gambling to refer to gambling that results in adverse consequences for gamblers, families, and communities [3]. Internationally, estimates of past-year problem gambling have ranged from 0.1% to 5.8% over the past decade [4]. Specifically, Australian and New Zealand national estimates suggest that past-year problem gambling affects 0.4% to 0.7% of adults, with a further 2% to 11% displaying moderate-risk gambling and 3.0% to 7.7% displaying low-risk gambling [5-7]. Despite relatively low prevalence estimates, problem gambling is associated with a high burden of harm [8], which can include financial strain and loss, relationship breakdown, emotional and psychological distress, health decline, cultural upset, reduced work or study performance, and social deviance [9]. Problem gambling is also highly comorbid with a range of mental health issues, including mood, anxiety, alcohol and substance use, and personality disorders [10-12].

### The Relapse Prevention Model

The relapse prevention model [13], a prominent and influential social-cognitive theory originally developed to explain relapse in substance use disorders, classifies factors or situations that can precipitate or contribute to relapse. Generally, these factors can be immediate determinants (high-risk situations, coping skills, outcome expectancies, and the abstinence violation effect) or covert antecedents that indirectly influence relapse (lifestyle imbalances, rationalizations, denial, apparently irrelevant decisions, and urges or cravings). A basic assumption of this model is that lapses are immediately preceded by a high-risk situation, broadly defined as any context that confers vulnerability to engaging in the target behavior, such as negative emotional states, interpersonal conflict, social pressure, testing of personal control, and nonspecific cravings. The model posits that positive outcome expectancies become particularly salient in high-risk situations, whereby the immediate positive effects of addictive behavior may be anticipated, and the possible delayed negative consequences of addictive behavior are ignored or discounted [13]. It also highlights that effective behavioral and cognitive coping in response to high-risk situations enhances self-efficacy, thereby reducing the probability of relapse [13,14].

The relapse prevention model has been reconceptualized [15] to emphasize the multidimensional, complex, nonlinear, and dynamic interaction among various precipitants that act jointly and interactively within high-risk situations to determine the likelihood of relapse. This model also incorporates the interaction among background factors (eg, years of dependence, family history, social support, and comorbid psychopathology), physiological states (eg, physical withdrawal), cognitive processes (eg, self-efficacy, outcome expectancies, craving, motivation, and abstinence violation effect), affective states, and coping skills. However, responding to a high-risk situation is related to both distal and proximal risk factors operating within both tonic processes and phasic responses. Tonic processes are distal risks or stable background factors that determine the *set point* or initial threshold for relapse. These processes, which indicate chronic vulnerability to relapse, often accumulate and lead to the instigation of a high-risk situation, providing the foundation for the possibility of relapse. In contrast, phasic responses are situational cognitive, affective, or physical states that can fluctuate across time and contexts and serve to activate lapses. Momentary coping responses can also serve as phasic events that determine whether a high-risk situation culminates in a lapse. The model predicts feedback loops, whereby lapse episodes can have reciprocal effects on the same factors (cognitive processes, affective states, and coping behavior) that contribute to the lapse. There is considerable empirical support for relapse prevention models across addictions [15,16].

In this model, cognitive processes that are relatively stable over time, such as outcome expectancies and global self-efficacy, are conceptualized as tonic processes, whereas cognitive processes that fluctuate over contexts and time, such as urges or cravings, as well as transient changes in outcome expectancies and self-efficacy, are conceptualized as phasic responses. Because it emphasizes the importance of nonlinear relationships and the timing or sequencing of events, the model does not articulate the temporal relationships between each of these cognitive processes. For example, a momentary reduction in self-efficacy in a high-risk situation could have a disproportionate influence on other cognitive processes, such as outcome expectancies [15]. There is emerging evidence of the role that cognitive processes play in gambling behavior and relapse as tonic processes; however, there is less evidence in relation to the role they play as phasic responses.

### Gambling Craving

Craving is a central phenomenon in addiction science. Despite the abundance of theoretical models, there is little consensus about its definition, etiology, and maintenance, and the terms craving and urge are often used interchangeably [17,18]. In the relapse prevention model, cravings are defined as the subjective desire to experience an appetitive target and urges are described as relatively sudden behavioral intentions or impulses to seek out and engage in an appetitive target [13,14]. This conceptualization is consistent with the integrative elaborated intrusion theory of desire [19,20], in which craving is defined as intense subjective desires for an appetitive target and urges are defined as specific desires for positive or negative reinforcement from an appetitive target [17]. Recent empirical studies attempting to delineate between gambling cravings and urges suggest that gambling craving is a higher-order and multifaceted construct, which is characterized by mental imagery, desire thoughts, and physiological sensations and triggered by various stimuli, including positive affect, negative affect, external cues, mental imagery, and desire thoughts [21]. In contrast, urges are a more narrowly defined construct comprising 2 core dimensions: intent and desire to gamble (due to expectations of positive reinforcement) and relief (due to expectations of negative reinforcement) [17].

Despite this conceptual confusion, the emerging cross-sectional literature highlights the important role that craving plays in the maintenance, exacerbation, and relapse of gambling problems. Specifically, findings suggest that gambling cravings are positively associated with problem gambling severity [22,23] and gambling relapse [24], negatively associated with abstinence [24,25], and are among the most frequent precipitants of relapse [26]. These findings are supported by qualitative research in which gambling cravings have been identified as a key construct associated with an increased risk of gambling relapse [27,28]. There is growing evidence that gambling cravings are relevant and useful intervention targets and potential mechanisms of change in both cognitive behavioral and mindfulness-based gambling interventions. Craving has predicted outcomes following cognitive behavioral treatment [29], and interventions that include craving management components have demonstrated efficacy in reducing cravings [27,28,30-43]. These studies typically targeted cravings using cognitive behavioral techniques, such as self-monitoring, psychoeducation, development of alternative responses, behavioral exposure exercises, and relapse prevention strategies, as well as mindfulness-based strategies such as urge surfing and guided breathing or body scan meditations.

### Gambling Self-efficacy

Self-efficacy, an important construct within social-cognitive theory, refers to feelings of confidence and capability to perform a behavior in a specific situational context to produce a desired outcome [44]. Addiction science has predominantly conceptualized self-efficacy in terms of perceived confidence to resist engaging in addictive behaviors in high-risk situations, but self-efficacy measures frame such resistance slightly differently, including confidence in *controlling* addictive behavior [45], *resisting the urge* to engage in addictive behavior [46], *avoiding* addictive behavior [47], *refusing* to engage in addictive behavior [48], or *abstaining* from addictive behavior [49]. Regardless of how resistance is framed, cross-sectional studies have consistently found that self-efficacy is negatively associated with both gambling behavior and problem gambling severity [22,23,45,47,49-53] and accurately discriminates between nonproblem and problem gambling samples [48,52]. Qualitative research supports these findings, suggesting that self-efficacy is a key construct in preventing relapse, which in turn increases motivation and commitment to maintain abstinence over time; however, the protective effect of self-efficacy weakens once relapse has occurred [27,28]. Similarly, there is some evidence that self-efficacy plays a protective role in preventing cravings from transitioning to gambling behavior but not when cravings are intense [23]. These findings highlight the potential of self-efficacy as an important intervention target and mechanism of change in treatment. Furthermore, self-efficacy has been demonstrated as an important predictor of treatment outcomes for gambling across several studies [54-56], and there is a small but growing body of literature reporting improvements in self-efficacy following interventions incorporating relapse prevention, cognitive behavioral, and motivational interviewing strategies [39,41,43,52,57-62].

### Positive Outcome Expectancies

Positive outcome expectancies are typically described as higher expectations or anticipation of the positive effects of future experience [13,14,44]. Theoretical conceptualizations suggest that outcome expectancies are associations among mental representations in long-term memory that are automatically activated under specific circumstances [63]. There is now growing cross-sectional evidence that global positive outcome expectancies [64-68] and specific positive outcome expectancies, such as financial, excitement, escape, ego enhancement, and social expectancies [69-78], are positively associated with problem gambling severity and related harm. Although few studies have explored the degree to which these expectancies change during treatment or are predictive of treatment outcomes, one study has found clinically and statistically significant reductions in global positive outcome expectancies from pre- to postresidential gambling treatment [79].

### Ecological Momentary Assessment of Cravings, Self-efficacy, and Positive Outcome Expectancies

These predominantly cross-sectional studies, which are subject to recall bias, treat cravings, self-efficacy, and gambling outcome expectancies as stable and enduring traits rather than transient or phasic states [80-82]. However, the reformulated relapse prevention model posits that transient changes in these cognitive processes can constitute phasic responses that interact with tonic processes and determine the likelihood of relapse [15]. Ecological momentary assessment (EMA), an event-level longitudinal methodology, overcomes the limitations of cross-sectional research by repeatedly measuring symptoms, emotions, behavior, and thoughts in real time and in natural environments [80]. Although there is now substantial EMA evidence that momentary cognitive processes (cravings, self-efficacy, and positive outcome expectancies) predict the occurrence of tobacco, alcohol, and substance use [83-89], few EMA studies have explored the associations between these processes and gambling behavior [90-92]. In the available studies, momentary cravings and self-efficacy, but not positive outcome expectancies, have predicted the likelihood of a subsequent gambling episode [90-92]. Moreover, all of these momentary cognitive processes constitute situational determinants of gambling behavior when they interact with other factors implicated in the relapse prevention model, such as high-risk positive reinforcement situations, self-efficacy, coping motives, cravings, positive emotional states, and coping styles [90-92].

### Just-in-Time Adaptive Interventions

These findings, which support the relapse prevention model, suggest that cravings, self-efficacy, and positive outcome expectancies constitute phasic precipitants of gambling behavior, although this may only occur for positive outcome expectancies when they interact with tonic precipitants, such as problem gambling severity [90]. The presence of these discrete but fluctuating precipitants, in combination with the complex and dynamic nature of gambling episodes or lapses, calls for the development of tailored interventions delivered in real time, such as just-in-time adaptive interventions (JITAIs). JITAIs are mobile health (mHealth) interventions that address dynamically changing individual needs by providing the type and amount of support required at the right time and only when needed [93-97]. They are *push* interventions, in which decisions about when and how support is provided are initiated by intervention protocols via computer algorithms rather than *pull* interventions initiated by individuals when they feel they require support [97,98]. mHealth interventions characterized by *just-in-time* (provision of the right type, timing, or amount of support) and *adaptive* (use of dynamic information from the individual to repeatedly select the type, timing, or amount of support) components have also been described as ecological momentary interventions, as long as they are dynamically and individually tailored [99].

The overall aim of JITAIs is to prevent negative health outcomes and promote the adoption and maintenance of positive health outcomes [94-98]. They are designed to provide support when individuals are in a *state of vulnerability* (a period of susceptibility to negative health outcomes) or a *state of opportunity* (a period of susceptibility to positive health behavior change), as well as a *state of receptivity* (able and willing to receive, process, and use the provided support) [94,95]. JITAIs identify how and when support should be offered by continuously monitoring dynamic internal states and ecological contexts in real time and in the natural environments of individuals using mobile and wireless technologies, including smartphone-embedded or wearable sensors and smartphone-delivered EMAs [93-96,98].

Nahum-Shani et al [94-96] have developed a comprehensive organizing scientific framework to guide the design of JITAIs. This framework describes the four key components that play an important role in JITAI design: (1) *decision points* (points in time at which intervention decisions are made), (2) *intervention options* (potential type, dose, timing, and delivery mode of support that can be delivered at any given decision point), (3) *tailoring variables* (data about the individual’s internal state or ecological context that is used to decide when and how to intervene), and (4) *decision rules* (a specification of which intervention option to offer, for whom, and when at each level of the tailoring variables). These components are guided primarily by the ultimate, long-term goal of the intervention (*distal outcome*) but also by the clearly defined near-time, short-term goals that the intervention is intended to achieve (*proximal outcomes*) [94]. JITAIs have been effective in supporting behavior change across a range of health behaviors, including addictive disorders, such as smoking, binge drinking, heavy drinking, and alcohol use disorders [93,94,96,99,100].

Similar to other mHealth interventions, JITAIs are characterized by high availability and accessibility, convenience, anonymity, portability, cost-effectiveness, and low burden, as well as the potential for real-world translation, scalability, and accurate data recording [93,97,99,101-103]. They also have the potential to reach underserved populations, including those who are unable or unwilling to participate in other interventions [99,101,102]. This is particularly important for gambling populations, given evidence that only a small proportion of people with problem and moderate-risk gambling (1 in 5 and 1 in 25 in Australia, respectively) access specialist face-to-face gambling services [104], despite an established evidence base indicating their efficacy [105-107]. These findings imply that face-to-face gambling treatment delivery does not provide sufficient access to evidence-based treatment [108]. The barriers to accessing face-to-face gambling treatment, which are now well-documented [109-111], include personal factors (eg, denial, shame, stigma, embarrassment, and a desire to deal with one’s own problem), resource limitations (eg, a lack of available services and trained clinicians), geographic inaccessibility, low awareness of treatment options, treatment costs, time commitments, childcare requirements, and reluctance to engage in treatments with a prespecified goal of abstinence. JITAIs overcome many of these barriers by leveraging mobile and wireless technologies to provide immediate, cost-effective, and low-burden treatment in moments of need.

Despite these clear benefits, the development of JITAIs targeting gambling behavior has been slow. Two smartphone apps that send notifications in response to the detection of proximity or entry into gambling venues by passive assessments using geolocation sensors to collect automated data (GPS, accelerometer, gyroscope, and magnetometer) have been developed: a smartphone-based problem gambling evaluation and technology testing initiative (*SPGeTTI*) [112] and *Don’t Go There* [113]. *SPGeTTI* also includes *pull* features that can be accessed on demand (self-monitoring gambling diary, relapse prevention tips, and help service contacts), whereas *Don’t Go There* allows an elected health professional to access the individual’s information. Despite low recruitment rates for a planned randomized controlled trial of *SPGeTTI*, focus group interviews revealed that gamblers reported high interest in the app. However, specific issues with *SPGeTTI* have been identified, such as excessive battery drainage. *Don’t Go There* is yet to be evaluated, with a usability study currently underway.

Two other gambling JITAIs that use active assessments via smartphone-delivered EMAs to collect data on internal states have been developed: *Jeu-contrôle* [114] and *GamblingLess: Curb Your Urge* [115,116]. Yet to be evaluated, *Jeu-contrôle* is a publicly available JITAI that uses EMAs to provide personalized feedback in relation to goal limits, with a view to supporting adherence to expenditure and time limits. In contrast, *GamblingLess: Curb Your Urge* is informed by the relapse prevention model and aims to reduce gambling cravings to prevent subsequent gambling episodes. This intervention, which was adapted from *GamblingLess*, an evidence-based web-based self-directed gambling program [41,43,115-120], tailors craving management activities to EMAs evaluating craving intensity and also provides these activities on demand. Key stakeholders rated the intervention content, helpfulness, acceptability, and usability highly and indicated that they would recommend the app to gamblers given its potential to increase gambling knowledge, attitudes, awareness, behavior change, intention to change, and help-seeking [115,116]. A pilot study of this JITAI [116] revealed promising findings, with more than a 70% reduction in the average number of gambling episodes and craving occurrences during the intervention period and a 10% decrease in momentary craving intensity immediately after a recommended intervention. There were also significant medium-to-large reductions in gambling symptom severity, gambling frequency, gambling expenditure, cravings, and self-efficacy at the postintervention and 1-month follow-up evaluations. At the 1-month follow-up evaluation, nearly half of the participants (10/21, 48%) reported recovery or improvement in the severity of gambling symptoms.

### Research Questions

This project aims to redress the gap in existing gambling service provision by evaluating a theoretically informed and evidence-based JITAI that builds on pilot data provided by the evaluation of *GamblingLess: Curb Your Urge* [115,116]. *GamblingLess: In-The-Moment* is a smartphone-delivered JITAI for people who want to quit or gamble less. It uses EMAs to collect comprehensive and accurate data on the dynamic cognitive processes articulated by the relapse prevention model. The JITAI uses *decision rules* specifying that individuals who are in a state of receptivity (available for treatment) and report a state of cognitive vulnerability characterized by high craving intensity, low self-efficacy, or positive outcome expectancies (*tailoring variables*) in EMAs sent during 3 semirandom times a day (*decision points*) are delivered tailored cognitive behavioral and third-wave interventions targeting these cognitive processes (*intervention options*). The intervention aims to reduce gambling symptom severity in the long term (*distal outcome*), and reduce the likelihood of gambling episodes (*primary proximal outcome*) in the short term via improved craving intensity, self-efficacy, and positive outcome expectancies (*secondary proximal outcomes*). The JITAI is intended for use as a stand-alone or adjunctive treatment during periods of active gambling behavior or as a relapse prevention tool during recovery.

A microrandomized trial (MRT), a form of sequential factorial design in which every participant serves as their own control, will be used to inform the optimization of this JITAI [93,98]. In this MRT, each participant will be randomized to a tailored intervention condition or no intervention control condition at each decision point across a 28-day period [121,122]. The primary aim of the MRT is to explore whether it is worthwhile to deliver a tailored intervention option at a time of cognitive vulnerability. Specifically, the aim is to explore whether, compared with the delivery of no intervention, the delivery of a tailored intervention reduces the probability of a subsequent gambling episode (primary proximal outcome) and improves craving intensity, self-efficacy, and positive outcome expectancies (secondary proximal outcomes). It is hypothesized that the delivery of a tailored intervention will be more effective than no intervention in reducing the probability of a gambling episode and improving craving intensity, self-efficacy, and positive outcome expectancies by the subsequent EMA. Should data allow, secondary exploratory research questions include the following:

1. *Which type of intervention option is most beneficial at a time of cognitive vulnerability?* Is the delivery of one intervention option (targeting cravings, self-efficacy, or positive outcome expectancies) more likely to reduce the probability of a subsequent gambling episode than the other intervention options?
2. *Under what conditions is the delivery of an intervention option most beneficial?* How do time-variant (EMA) factors (time of day, time of week, craving intensity, self-efficacy, positive outcome expectancies, psychological distress, impulsivity, subjective alcohol intoxication, readiness to change, gambling availability [financial and location], and social context) and time-invariant (preintervention survey) factors (gambling symptom severity, gambling frequency, gambling expenditure, gender, and age) influence the intervention effect on the probability of a subsequent gambling episode?
3. *How do the proximal effects of intervention options change over time as the treatment progresses?* How does the effect of a tailored intervention on the probability of a subsequent gambling episode change over the course of the 28-day MRT?

## Methods

### Ethics Approval

This trial has been approved by the Deakin University Human Research Ethics Committee (2020-304) and registered with the Australian New Zealand Clinical Trials Registry (ACTRN12622000490774).

### Trial Design

MRTs have significant advantages over randomized controlled trials as participants act as their own control group, providing a strong capacity for causal inferences and increased power to detect treatment effects [98]. Moreover, they are designed to facilitate the optimization of JITAIs, which involves determining how a JITAI should be adjusted to make it more effective, efficient, and scalable [98,122,123]. Participants will participate in a 28-day MRT, in which they will be prompted via push notifications on their smartphones to complete a time-based EMA 3 times daily (*decision points*). In this EMA, *tailoring variables* used to determine intervention eligibility include momentary craving intensity, self-efficacy, and positive outcome expectancies. *Decision rules* based on EMA item cut points will determine eligibility for a tailored intervention, which could consist of a craving, self-efficacy, or positive outcome expectancy *intervention option*. Participants will be randomly allocated to either a tailored intervention condition or no intervention control condition at each *decision point* across the 28-day trial period. To maintain the integrity of the MRT evaluation, the JITAI will be evaluated as an entirely *push* intervention during the 28-day MRT period [97,98]. This trial will provide important empirical data that can be used to facilitate the optimization of the JITAI to make it a more effective, efficient, and scalable intervention.

**Figure 1:**
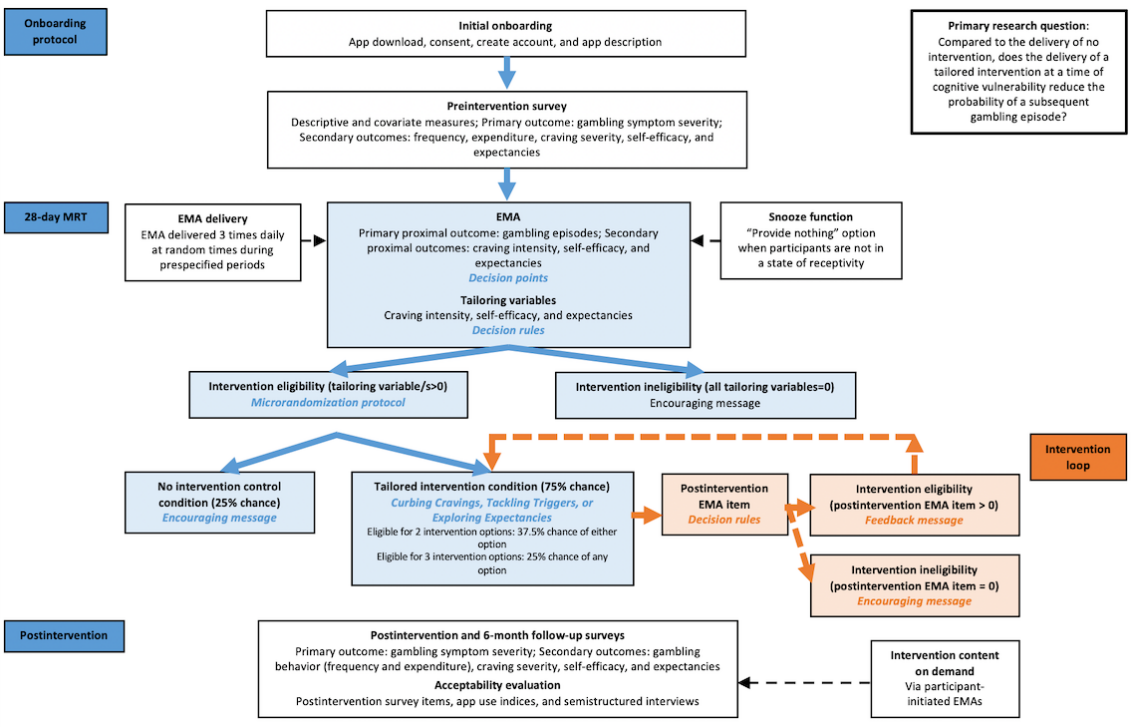
Overview of the *GamblingLess: In-The-Moment* trial design. EMA: ecological momentary assessment; MRT: microrandomized trial.

The MRT will be supplemented with (1) a within-group follow-up evaluation to explore the long-term outcomes of the intervention in relation to the primary (gambling symptom severity) and secondary (gambling frequency, gambling expenditure, cravings, self-efficacy, and positive outcome expectancies) outcomes from the preintervention evaluation to the postintervention and 6-month follow-up evaluations as well as the predictors of long-term treatment outcomes and (2) an evaluation of the acceptability of the JITAI using postintervention surveys, app use and engagement indices, and semistructured interviews. An overview of the trial design is shown in Figure 1.

### Participant Recruitment and Reimbursement

Participants will be recruited across Australia and New Zealand, using a range of strategies, such as web-based advertising (eg, Google Adwords), social media (eg, Facebook and Instagram), gambling-related websites (eg, GambleAware, Gambling Helpline, Gambling Help Online, and Australasian Gaming Council), and advertisements in public places (eg, universities, general practices, health services, mental health services, and alcohol and other drug services). Gambling counseling services and gambling venues may also be requested to assist with participant recruitment. The eligibility criteria will include (1) current Australian or New Zealand residence, (2) ≥18 years of age, (3) installation of the app from an internet-enabled smartphone, (4) willingness to receive notifications from the app, (5) fluency in the English language, and (6) seeking support for one’s own gambling. The target population for *GamblingLess: In-The-Moment* comprises people who want to quit or gamble less. Consistent with a pragmatic design, the intervention will be available to any interested gambler, regardless of the level of gambling symptom severity or whether they are seeking other forms of support or treatment [124]. Moreover, consistent with a harm minimization approach, participants can select abstinence or nonabstinence treatment goals [125-127]. Participants in the trial will be compensated for their time in e-gift vouchers: US $0.70 for each EMA completed and a US $14 bonus if >75% of the EMAs are completed (to a maximum of US $70), US $35 for completion of the postintervention survey, US $50 for completion of the 6-month follow-up survey, and US $35 for the optional semistructured interview.

### Onboarding Protocol

Participants will be required to download *GamblingLess: In-The-Moment* from app stores and provide explicit agreement to the trial plain language statement, as well as the app platform’s terms of use and privacy policy. By agreeing to the terms and privacy policy, participants declare that they have read and understood the plain language statement, are freely participating in the trial according to this statement, meet the eligibility criteria, and understand their privacy rights. They will be required to create an account on the platform by providing the requisite details for the app platform (username, email address, password, and display name). The in-app onboarding protocol will then require participants to read a brief app description and information about how to use the app. They will then be required to indicate whether they are gamblers, family members, or stakeholders in order to complete the preintervention survey (see *Within-Group Follow-up Evaluation*). Finally, they are required to record their mobile number, indicate their interest in being contacted for further research, and indicate their interest in participating in the semistructured interviews. Following this onboarding protocol, the participants will be encouraged to complete their first EMA.

### Distal and Proximal Outcomes

The *distal outcome* of *GamblingLess: In-The-Moment* is the severity of gambling symptoms. The *primary proximal outcome* is reduced probability of a subsequent gambling episode (measured at the subsequent EMA). This outcome will be measured in the EMA using the item, “Have you gambled since the last time you checked in?” with a binary response option (yes or no). The secondary proximal outcomes are improvements in subsequent craving intensity, self-efficacy, and positive outcome expectancies (measured at the subsequent EMA).

### EMA Features

#### Overview

*GamblingLess: In-The-Moment* will use an EMA protocol (Multimedia Appendix 1) employing in-app time-based sampling (ie, semirandomly prompting individuals to input internal states and ecological contexts), which will incorporate event-based sampling (collecting data around specific and discrete gambling episodes). Each EMA, which will take approximately 1 minute to complete, will comprise 10 items measuring momentary internal states and ecological contexts (including the tailoring variables), most of which are recorded on varying 5-point response scales presented in a multiple-choice format. To ensure an accurate record of gambling events, participants will also be administered an event record (Multimedia Appendix 1) in each EMA, in which they will record gambling episodes (primary proximal outcome) and associated expenditure using single items that have been used in previous EMA and ecological momentary intervention gambling research [90,92,115,116].

#### Decision Points

During the 28-day MRT, participants will be prompted via push notifications to complete an EMA delivered through the app 3 times daily at random times during the prespecified periods: morning (8:30 AM-11 AM), afternoon (1 PM-3:30 PM), and evening (5:30 PM-8 PM; Figure 1). At each EMA notification, participants can auto-launch the EMA via the notification or app. Participants will be allowed 2 hours to complete an EMA to preserve the momentary nature of the intervention while accommodating the potential for possible unavailability (eg, driving and working) of the participant at the initial prompt time [98,128].

#### Tailoring Variables

The tailoring variables for *GamblingLess: In-The-Moment* include momentary craving intensity, self-efficacy, and positive outcome expectancies measured during each EMA (Multimedia Appendix 1). The craving item was adapted from the first item of the Gambling Symptom Assessment Scale [129], the self-efficacy item was adapted from the Brief Situational Confidence Questionnaire-Gambling [130], and the positive outcome expectancy item was adapted from the Gambling Outcome Expectancy Scale [69]. The JITAI will tailor the type, timing, and amount of support to individual needs [94-96,99,100] (Figure 1). The tailoring variable data received from each participant will be used to individualize treatment by repeatedly selecting the *type* of intervention content (craving, self-efficacy, or positive outcome expectancies). The flexible collection of tailoring variable data will allow for the *timely* individualization of intervention options at specific moments when individuals are especially in need of support but not when they do not need support or are unreceptive to support. Finally, the dosage or *amount* of support will be tailored to individual needs via an intervention loop, whereby participants who continue to require support after completing an intervention activity will be offered additional support.

#### Additional EMA Items

Additional single items measuring other momentary internal states and ecological contextual factors highlighted by the relapse prevention model [13,15] will be included in each EMA to explore the conditions under which the JITAI is most effective: psychological distress (based on the distress thermometer: Psychosocial Distress Practice Guidelines) [131], readiness to change (based on the gambling readiness ruler) [132], subjective alcohol intoxication (based on the Subjective Effects of Alcohol Scale) [133], impulsivity (based on an EMA item from the Momentary Impulsivity Scale) [134], social context (based on an EMA item measuring social context for alcohol use) [135], financial gambling availability (based on an EMA item assessing money for preferred products) [136], and location gambling availability (based on an EMA item assessing cigarette availability [137]) (Multimedia Appendix 1).

#### Welcome and Reminder Protocol

The following contact protocol will be adopted to enhance EMA compliance during the 28-day MRT: (1) an automated welcome email to all participants following onboarding, (2) a reminder email to participants who fail to complete onboarding or fail to complete an EMA for more than 48 hours following onboarding, and (3) a reminder telephone call by clinically or qualitatively trained research fellows to participants who still fail to complete onboarding or an EMA in the subsequent 48-hour period (with a follow-up SMS message if no answer or second follow-up email if no valid phone number was provided). Participants who fail to complete an EMA following this protocol will receive no further contact but (as long as they complete onboarding and at least one EMA and have some engagement with app-intervention activities) will be contacted to request completion of the postintervention and 6-month follow-up surveys.

### Intervention Options

#### Tailored Intervention Condition

The intervention options in *GamblingLess: In-The-Moment* are informed by the relapse prevention model [13,15] as well as data from the *GamblingLess* program of research [41,43,115-120]. The intervention options were designed to target the cognitive processes that mark a state of cognitive vulnerability (cravings, low self-efficacy, and positive outcome expectancies) (secondary proximal outcomes) for the occurrence of a gambling episode (primary proximal outcome). The resulting program comprises 53 activities across three tailored intervention options (modules): (1) *Curbing Cravings*, (2) *Tackling Triggers*, and (3) *Exploring Expectancies*. Consistent with more recent conceptualizations of coping strategies explicated by the relapse prevention model, these interventions include cognitive, behavioral, and third-wave (mindfulness and acceptance) strategies [13,15,138]. Cognitive behavioral treatments have the most established evidence base in both face-to-face and mobile treatment of problem gambling [105-107,139,140], with mindfulness-based interventions receiving emerging empirical support [141,142]. The activities in each of these modules are displayed in Multimedia Appendix 2.

The intervention activities were selected based on their appropriateness for repeated delivery, effectiveness in previous research, and previous acceptability feedback [41,43,115,116]. The selected activities were developed for smartphone delivery with a focus on engagement, interactivity, user preferences, participant literacy, inclusiveness, and ease of use, with the aim of encouraging autonomy and creating an aesthetically pleasing design. Activities involved user interaction and gamified using multimedia delivery strategies comprising a combination of video activities, audio activities, personalized feedback, quizzes, open-ended items, and multiple-choice items. All video-based activities were publicly sourced videos from YouTube, with written permission obtained from each creator. To assist participants in selecting an activity appropriate to their current environment or social situation, all activities are labeled as text, video, interactive, audio, or text and image on each menu. Consistent with the pilot study, *GamblingLess: Curb Your Urge* [115,116], most activities take <5 minutes to complete.

#### Intervention Option 1: Curbing Cravings

The *Curbing Cravings* intervention option includes a bank of 10 craving management activities, including psychoeducation, distraction, breathing exercises, progressive muscle relaxation, mindfulness meditation, urge surfing, imagery, cognitive reframing, decisional balance, and planning [13,14]. In the *GamblingLess* trial, participants reported the lowest self-efficacy for high-risk situations related to urges and temptations [41,43]. Despite the delineation between craving and urges described earlier, the term urges was used in all user-facing aspects of the intervention (ie, EMA items and activity content; the intervention title is not user-facing), given anecdotal evidence in the addiction field that this is the most understandable, accessible, and commonly used term in addiction science [143-145].

#### Intervention Option 2: Tackling Triggers

The *Tackling Triggers* intervention option contains 25 activities to improve self-efficacy across 5 types of high-risk situations: financial pressure, unpleasant emotions, social pressure to gamble, testing control over gambling, and conflict with others. Participants in the *GamblingLess* trial [41,43] reported the lowest self-efficacy in these situations (with the exception of urges and temptations, which are targeted separately in the *Curbing Cravings* intervention option). Each situation type comprises a bank of 5 activities designed to increase a sense of competency and mastery by teaching participants to identify, anticipate, plan for, and effectively cope with these high-risk situations [13,14]. These include behavioral (eg, self-monitoring, goal-setting, behavioral substitution, progressive muscle relaxation, psychoeducation, assertiveness training, conflict resolution training, lapse management, and planning), cognitive (eg, cognitive reframing, imagery, and decisional balance), and acceptance or mindfulness (eg, cognitive defusion and mindfulness meditation) strategies [13,14].

#### Intervention Option 3: Exploring Expectancies

The *Exploring Expectancies* intervention option contains 18 activities to redress 3 types of positive outcome expectancies: excitement, escape, and money. These positive outcome expectancies have consistently displayed positive associations with problem gambling severity and gambling-related harm, particularly in Australian adult samples [69,75,76]. Consistent with the relapse prevention model [13], the focus of intervention activities in this intervention was to explore the validity and reality of positive outcome expectancies by contrasting the possible immediate positive consequences with the delayed negative consequences of gambling [146]. Meta-analytic evidence supports the efficacy of such expectancy challenge interventions for alcohol abuse prevention [147]. Each type of expectancy comprises 6 activities, including behavioral (eg, self-monitoring, personalized normative feedback, psychoeducation, progressive muscle relaxation, and behavioral substitution), cognitive (eg, decisional balance, imagery, and expectancy challenging), and third-wave (eg, mindfulness meditation, and cognitive defusion) activities to redress these positive outcome expectancies.

#### No Intervention Control Condition

In the MRT, the no intervention control condition involves participants being delivered a brief encouraging message, after which their interaction with the app will end. This tailored message involves acknowledgment that participants have an urge, low self-efficacy in high-risk situations, or positive outcome expectancies, as well as encouragement to consider doing something to reduce their urge, avoid or cope with high-risk situations, or reduce their expectations. In this condition, the participants are not provided with any intervention activities.

### Decision Rules

The decision rules are illustrated in Figure 1. At each decision point during the 28-day MRT, responses to EMA items assessing craving intensity (tailoring variable 1), self-efficacy (tailoring variable 2), and positive outcome expectancies (tailoring variable 3) are used to determine eligibility for the delivery of the tailored intervention, according to a set of predetermined decision rules (scoring >0 on each tailoring variable). At each decision point, participants who do not exceed the cut point for any of the tailoring variables are not eligible for any intervention. These participants will receive a brief encouraging message and their interaction with the app will end. In contrast, participants who exceed the cut point on one or more of the tailoring variables are eligible for a tailored intervention (ie, curbing craving, tackling triggers, *or* exploring expectancies).

### Microrandomization Protocol

The microrandomization procedure will then be applied, whereby eligible participants will be microrandomized to a tailored intervention condition or no intervention control condition (Figure 1). Overall, the microrandomization procedure will involve eligible participants having a 75% chance of being microrandomized in the tailored intervention condition and a 25% chance of being microrandomized in the no intervention control condition. However, because the reformulated relapse prevention model does not presume that certain factors are more influential than others [15], participants exceeding the cut point on more than one of these tailoring variables will be randomly allocated to a relevant intervention option (curbing cravings, tackling triggers, or exploring expectancies). Specifically, participants who are eligible for 2 intervention options will have a 37.5% chance of receiving either intervention option, and those who are eligible for 3 intervention options will have a 25% chance of receiving any of the intervention options. This microrandomization protocol is fully automated, which guarantees that the administration of treatments and assessment of outcomes are blinded.

Following completion of each EMA, participants who are microrandomized to the no intervention control condition will be sent an encouraging message, and their interaction with the app will end. Participants microrandomized to the tailored intervention condition will be sent to the relevant intervention dashboard, which comprises a menu of intervention activities from which they can select. Specifically, participants who are allocated to the craving intervention will be taken to the craving intervention dashboard and asked to select an intervention activity. In contrast, participants who are allocated to the self-efficacy and expectancies interventions will be asked to select a specific type of trigger or expectation, administered an EMA item specific to their selected trigger or expectation (Multimedia Appendix 3), then taken to the relevant intervention dashboard, and asked to select an intervention activity.

### Intervention Loop

Following the completion of an intervention activity, participants are asked to complete the specific EMA item associated with the intervention group to which they were allocated (postintervention EMA item; Multimedia Appendix 3). Their response to the postintervention EMA item is then subjected to the same decision rules used for the time-based EMA. Participants who fail to reach the cut point on this postintervention EMA item will be presented with an encouraging message, and their interaction with the app will end. Participants who exceed the cut point (ie, score one or more) on the specific EMA item will be presented with a feedback message in which their response to their postintervention EMA item is compared with their time-based EMA response on the same item, encouraged to select another intervention activity, and returned to the relevant intervention dashboard. This intervention loop continues until the participant fails to exceed the cut point or closes the app (Figure 1). At several locations within the app, as well as in the welcome email and trial plain language statements, participants are informed that they can stop the loop at any time by closing the app to ensure that they do not adjust their response to break the loop.

### *Provide Nothing* Option

Importantly, a *provide nothing* option is provided for situations in which the participant is unreceptive, support is not required, or the provision of support may be unsafe, inconvenient, or unethical [94-96,98]. Specifically, support will not be offered if participants ignore the push notification prompting EMA completion or press the *snooze* function to indicate that they are currently unable to complete the EMA (which suggests that they are not in a state of receptivity).

### Other App Features

The home dashboard includes quick links to the *Check In Here*, *Get More Support*, and *More* features. The *Check In Here* quick link allows participants to complete an EMA within the allowed 2-hour period and provides an encouraging message when participants attempt to complete an EMA if more than 2 hours have passed since the last notification. The *Get More Support* quick link, which is also available on each of the intervention activity menu dashboards, provides click-to-call and click-to-email functions to Australian and New Zealand helpline and web-based specialist gambling services. This feature allows participants to escalate the support they receive, including immediate crisis support [101]. The *More* quick link provides information about the app, the trial, contact details, the platform’s privacy policy, the plain language statement, profile information, account details, and sign out. Other app features include the *Did You Know?* feature, which delivers brief passive psychoeducational messages related to cravings, self-efficacy, and positive outcome expectancies before the delivery of every intervention activity and the *Pick For Me* feature on each intervention activity menu, whereby the app randomly selects one of the intervention activities on the menu for participants.

### Within-Group Follow-up Evaluation

A within-group evaluation of outcomes over a 6-month follow-up period will supplement the MRT to (1) evaluate within-group change over a longer period and (2) explore predictors of longer-term treatment outcomes (including app use over the 6-month follow-up period). Although the preintervention survey will be automated via the app before beginning the 28-day MRT period, participants will be prompted by email to complete the postintervention and follow-up surveys via Qualtrics (Qualtrics XM). Descriptive and covariate measures will include participant type (gambler, family member or friend, and clinician, researcher, or policy maker), sociodemographic characteristics, problem gambling activities, intended gambling behavior (measured using the Timeline Follow-Forward, a novel adaptation of the Timeline Follow-Back [148]), and other help-seeking (measured using the Help Seeking Questionnaire [149]). The primary outcome will be gambling symptom severity (measured using the Gambling Symptom Assessment Scale [129]), and secondary outcomes will include gambling frequency and expenditure (measured using a timeline follow-back at the preintervention evaluation, the EMA event record data at the posttreatment evaluation, and single items at the 6-month evaluation) and the cognitive processes targeted by the intervention: craving severity (measured using the Penn Gambling Craving Scale [25]), self-efficacy (measured using the Brief Situational Confidence Questionnaire-Gambling [130]), and positive outcome expectancies (measured using the Excitement, Escape, and Money subscales of the Gambling Outcome Expectancies Scale [69]; Multimedia Appendix 4). Each evaluation will be completed in approximately 10 to 15 minutes. Ideally, follow-up evaluation surveys will also be conducted 12 and 24 months after the intervention, but this will be dependent on obtaining additional funding.

A follow-up protocol will be implemented to enhance the completion of the postintervention and 6-month follow-up surveys: (1) an email requesting survey completion, (2) a reminder email requesting survey completion within a week to participants who fail to complete the survey in the subsequent 1-week period, and (3) up to 2 reminder telephone calls by clinically or qualitatively trained research fellows to participants who fail to complete the survey in the subsequent 3-week period (with a follow-up SMS text message if no answer or a further follow-up email if no valid phone number was provided). An advance notice email will also be sent 1 week before the 6-month surveys are sent. At each time point, the option to complete the survey over the phone with a trained research fellow will be offered. Participants who fail to complete the surveys following this protocol will receive no further contact, but participants failing to complete the postintervention survey will be contacted to request the completion of the 6-month follow-up survey.

During the 6-month follow-up evaluation period, tailored intervention content will be available to participants on demand. Although Nahum-Shani et al [94] defines JITAI designs as a *push* intervention approach, participant-determined features may accommodate conditions in which participants know when and what type of support is required, promote autonomy by facilitating agency and control, reduce waste and disruption, generalize learned skills, and maintain therapeutic gains [94,97-99,101,150]. During this period, participants will not receive push notifications to complete EMAs and the microrandomization protocol will not be applied (ie, all participants will be allocated to the tailored intervention condition, with the no intervention control condition turned off). However, they will be able to access tailored intervention content via participant-initiated EMAs (ie, they can complete an EMA at any time, which will direct them to tailored intervention content). This approach has been adopted to encourage participants to incorporate coping skills in their everyday lives when they recognize states of vulnerability or opportunity and are motivated to initiate access to support [97-99,101]. The degree to which app use across the 6-month follow-up period influences longer-term treatment outcomes will be explored.

### Acceptability Evaluation

The acceptability of *GamblingLess: In-The-Moment*, operationalized as a multifaceted construct that reflects the extent to which participants consider the intervention to be appropriate based on the cognitive and emotional responses they have to the intervention [151], will be explored using multiple methodologies. *Postintervention surveys* will evaluate the subjective quality and perceived impact of the JITAI using the 4-item subjective quality and 6-item perceived impact subscales of the Mobile App Rating System [152], as well as the perceived helpfulness of additional features (eg, in-person support, web-based discussion boards, motivational messages, feedback, and push and pull features), EMA frequency, and program duration. *App use and engagement indices* will be used across the 28-day MRT and 6-month follow-up period to explore download information, onboarding information, app use information (eg, EMA compliance, intervention eligibility, intervention compliance, participant retention, and intervention activities selected), and the use of specific app features (eg, intervention loop, *Pick For Me*, and *Get More Support*). *Semistructured interviews* will be conducted with a minimum of 10 participants from the MRT to explore the reasons for using the app, program duration, EMA frequency and timing, perceived helpfulness of the intervention activities and specific features, perceived helpfulness of additional features, impact on behavior change, the app’s look and feel, and areas for improvement. Given the funding source, participants from New South Wales will be prioritized and stratified according to gender and app use. These interviews, which will be conducted at the end of the 28-day MRT, will be conducted by clinically or qualitatively trained research fellows via video conferencing or telephone. Interviews will be audio recorded for transcription and data analysis purposes, and data will be analyzed using thematic analysis at a semantic level based on the guidelines by Braun and Clarke [153].

### Statistical Analyses

To assess the research questions, the method of generalized estimating equations (GEE) will be used, with an appropriate link function for the outcome of interest (eg, identity and logit). Although the intention is to use an exchangeable working correlational structure for analyses, alternative correlational structures based on the observed within-person correlation pattern over the course of the study (eg, independent or autoregressive) will be considered. For all MRT analyses, the (lagged) outcome of interest (eg, gambling episode at Time_t+1_) will be regressed on to a variable denoting the intervention received (eg, intervention options 1, 2, and 3 or no intervention) at Time_t_, as well as covariates (including unbalanced time, time since prior assessment, and other forms of help-seeking). To assess the primary research question and secondary research question 1, the analyses will examine the effects of the tailored intervention versus no intervention control on the probability of a subsequent gambling episode, stratified analyses focusing on each intervention option separately versus no intervention control (on the specific outcome related to each intervention option; ie, craving intensity, self-efficacy, and positive outcome expectancies), and formal tests comparing the magnitude of each intervention with the no intervention condition on the probability of a subsequent gambling episode. Secondary research questions 2 and 3 will be examined by specifying the interaction terms between the intervention variable and the interaction variable of interest (eg, psychological distress and time). Consideration will be given to making appropriate adjustments in line with modern causal methods for assessing effect moderation [154].

To explore the long-term outcomes of the intervention (within-group follow-up evaluation), distal outcomes will be assessed using GEE by regressing the outcome of interest (eg, gambling symptom severity) on a variable denoting time (ie, preintervention, postintervention, 6-month follow-up variables) and covariates. The factors associated with longer-term treatment outcomes will also be explored using GEE by regressing the outcome of interest (eg, clinically significant change in gambling symptom severity) with selected preintervention, postintervention, and app use variables (eg, the number of participant-initiated EMAs completed in the 6-month follow-up period). Where appropriate, missingness will be addressed using multiple imputation, with appropriate accounting for the multilevel nature of the data (eg, see multilevel multiple imputation [155]).

### Clinical Significance

Supplementary analyses will consider the clinical significance of any effect (ie, meaningful changes in the participant’s life) [156]. Effect sizes will be calculated for all primary and secondary outcomes. These group-level examinations of effectiveness will also be supplemented by metrics of individual-level change for all primary and secondary outcomes. A reliable change index will be used to assess changes beyond those attributable to chance or measurement error [157]. Clinically significant change, as outlined by Jacobson and Truax [156], will subsequently be calculated using functional score ranges where possible (Gambling Symptom Assessment Scale score of ≤20) or a convention of at least a 25% change in scores in the positive direction [158]. At the posttreatment and 6-month follow-up evaluations, each participant’s status will be defined as *recovered* (final score falls into the functional range and corresponds to a reliable change), *improved* (final score falls into the dysfunctional range and corresponds to a reliable change), *unchanged* (final score does not correspond to a reliable change), or *deteriorated* (final score corresponds to a relative change in the negative direction).

### Sample Size

The project aims to recruit a sample of 200 participants. A sample size of 120 participants would provide >80% power to detect a small true intervention effect size for the primary binary outcome (ie, gambling episode) of relative risk=1.21 (at Cronbach α=.05; availability parameter=0.3; randomization *P*=.25; outcome without intervention *P*=.25) [159,160]. In *GamblingLess: Curb Your Urge*, attrition was 39% when considering participants who completed the baseline measures and 19% when considering participants who downloaded the app after completing the baseline measures [116]. As such, this project will provide sufficient power to detect true effects even under a conservative attrition rate of 40% from the original sample of 200 participants at baseline.

## Results

### Development of GamblingLess: In-The-Moment

**Figure 2:**
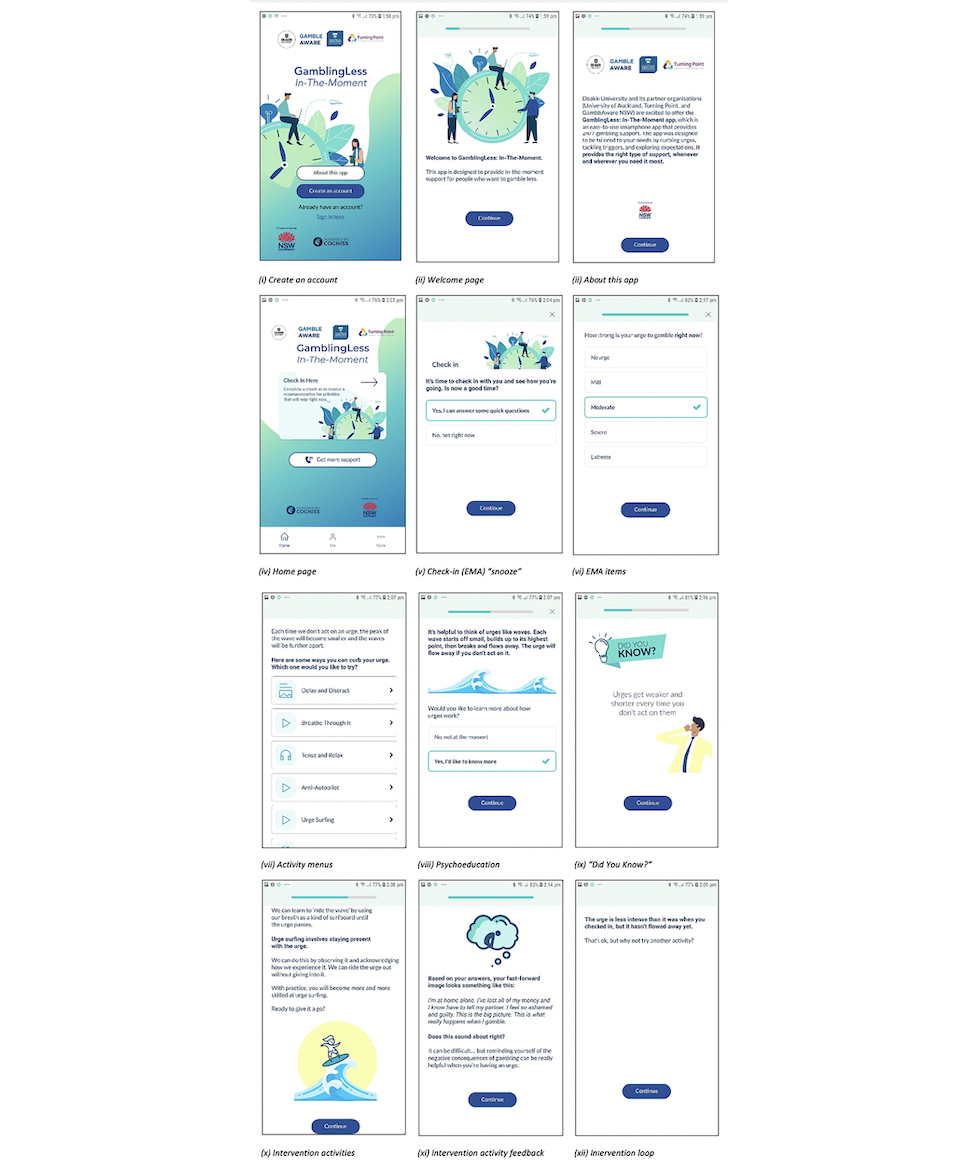
Illustrative screenshots of *GamblingLess: In-The-Moment*.

Development and evaluation of *GamblingLess: In-The-Moment* was funded in June 2019 by the New South Wales Government’s Responsible Gambling Fund. The development of *GamblingLess: In-The-Moment* involved a multidisciplinary team comprising behavior change expertise from clinical and social psychology, implementation science, biostatistics and research design, and technology developers, consistent with recommendations for technology development [94]. *GamblingLess: In-The-Moment* is part of a suite of theoretically informed and evidence-based web-based and mobile gambling interventions (*GamblingLess*). The development of the treatment content was led by the NAD, a clinical psychologist, and the app was hosted on the Cogniss behavior change platform, which was created by 2and2, specialist developers of custom tech solutions for learning, health, and behavior change. Illustrative screenshots of JITAI are displayed in Figure 2.

### User Testing of GamblingLess: In-The-Moment

Following its development, *GamblingLess: In-The-Moment* was subjected to user testing with 13 gambling stakeholders from June 2021 to July 2021, including 5 gambling clinicians, 5 gambling researchers, and 3 gambling consumers, who had scores in the problem gambling range of the Problem Gambling Severity Index (mean 18.7, SD 10.6). These user-testing participants comprised 5 men and 8 women, who tested the app on both Android (4/13, 31%) and Apple (9/13, 69%) devices. They were reimbursed with a US $35 e-gift voucher to download the app, create an account, test that the app functions as intended over a 3-day period, and evaluate the acceptability of the app via a Qualtrics XM survey (Multimedia Appendix 5). Participants completed the Mobile App Rating Scale [152], which comprises 23 items across 4 subscales measuring the overall quality of the app, the subjective quality of the app, and the perceived impact of the app [152].

Participants rated the user-testing version of *GamblingLess: In-The-Moment* over a minimum acceptability score of 3 for all Mobile App Rating Scale subscales, suggesting that the app can be recommended for reducing gambling symptom severity [161]. All tailored interventions were rated highly in terms of ease of completion (>8 out of 10) and helpfulness (>7 out of 10). Users also rated specific features highly (out of 10), particularly the helpfulness of the *Click to Call/Email* (mean 8.9, SD 1.3), *Pick for Me* option (mean 8.6, SD 1.8), and *Did You Know?* messages (mean 8.5, SD 1.3). Qualitative data were also generally positive, with positive comments relating to comprehensive, accurate, and concise information; ease of use; strategy range; helpfulness, practicality, and interactivity; the customization of feedback based on participant responses; the use of accessible and respectful language; the look and feel of the app, including graphics, multimedia, and interactivity; and the *Pick For Me* function. Participants generally indicated that the app would be a great resource, both as a stand-alone and adjunct intervention. Although few content or flow issues were identified, participants commented on several technical issues, predominantly in relation to notification frequency, the functionality of the deep link from the notification to the EMA, the accuracy of the check-in duration windows, and loading times (particularly in relation to Timeline Follow-Back and Timeline Follow-Forward calendars). These issues were resolved before the app was released for evaluation.

### Trial Progress

*GamblingLess: In-The-Moment* is available for download during this trial on Apple (App Store) and Android (Google Play Store) devices. Following advertising on March 22, 2022, a total of 3 pilot participants were recruited to ensure that the app and evaluation protocols functioned as intended. Advertising for the trial began on March 29, 2022. As of June 24, 2022, a total of 84 participants had been recruited for the trial. The results are expected to be published mid- to late-2024.

## Discussion

### Overview

This project aims to redress the gap in existing service provision by developing and evaluating a theoretically informed and evidence-based JITAI for gamblers who want to quit or gamble less. Consistent with JITAI development recommendations [94], *GamblingLess: In-The-Moment* was developed by a multidisciplinary team involving clinical and social psychology, implementation science, biostatistics and research design, and technology development. It uses *decision rules* specifying that individuals who are in a state of receptivity (available for treatment) and report a state of cognitive vulnerability characterized by high craving intensity, lowered self-efficacy, and positive outcome expectancies (*tailoring variables*) in time-based EMAs sent 3 semirandom times a day (*decision points*) are delivered tailored cognitive behavioral and third-wave interventions targeting these cognitive processes (*intervention options*). The JITAI will tailor the type, timing, and amount of support for individual needs.

The evaluation of the JITAI will involve a 28-day MRT, in which the JITAI will be evaluated as an entirely *push* intervention approach. Information from the MRT will be used to optimize *GamblingLess: In-The-Moment* to make it a more effective, efficient, and scalable intervention. This trial will provide important empirical data to identify more refined decision rules specifying which intervention options should be delivered as well as to explore when and for whom the intervention is effective [97,98]. This may involve discarding less effective or more burdensome options, delivering the intervention during specific internal states and ecological contexts, or modifying the timing and cut points for each tailoring variable [96-98].

Evaluations of JITAIs, particularly those using MRTs, generally preclude evaluations of long-term outcomes. Therefore, MRT will be supplemented with a 6-month within-group follow-up evaluation to assess and predict long-term outcomes. During this 6-month period, participants will be able to access the intervention content on demand via participant-initiated EMAs when they recognize states of vulnerability or opportunity and are motivated to initiate access to support. Unlike many other JITAI evaluations [99], within-group follow-up evaluations will also facilitate the consideration of the clinical impact of *GamblingLess: In-The-Moment* in addition to the statistical significance of the findings.

Consistent with recommendations [99,162], the development of *GamblingLess: In-The-Moment* used an iterative and user-centered approach to its design. The intervention has been subject to stakeholder user testing, which suggests that the JITAI is an acceptable gambling intervention, and subsequent consideration of user-testing feedback. The acceptability of the intervention will also be explored in the trial, using both qualitative and quantitative methods. This information can be used to evaluate participants’ perceptions related to the app’s subjective quality and perceived impact. It will also inform the future development of the app by providing information relating to individual intervention activities, the app’s specific features, and additional features that could be included in future iterations of the app. For example, it may be that participants indicate a preference for a more traditional *pull* approach, in which they initiate intervention access when they require support, or the addition of some *participant-determined* features or *on-demand* intervention content to this JITAI [94]. The addition of such features may accommodate situations in which individuals recognize states of vulnerability, thereby maintaining therapeutic gains by encouraging coping skills in everyday life, enhancing generalization of learned skills, promoting autonomy by facilitating agency and control, and involving less disruption and burden [94,97-99,150]. Similarly, participants may indicate a preference for the addition of human support via the involvement of clinicians, guides, coaches, or peers [103,162-164] or digital avatars in the form of personal coaches and assistants [165], which may enhance motivation, engagement, and adherence to the requirements of the intervention [163]. The acceptability evaluation will also offer the opportunity to explore participant preferences for program duration, as well as the frequency and timing of EMAs. For example, there is a risk that 3 EMAs each day are an obstacle to sustained engagement or that restricting the timing of the EMAs to daytime hours is not aligned with high-risk situations occurring outside these hours. This information is therefore particularly important to inform the limited evidence base regarding transitory changes in the presence of urges or cravings, self-efficacy in high-risk situations, or positive outcome expectancies.

This study will be one of the first to examine the effectiveness of real-time support for reducing gambling behavior and the first to achieve this by comprehensively addressing the cognitive processes underlying gambling lapses and relapses, as articulated by the highly influential relapse prevention model. Given that JITAI development and evaluation of gambling problems is an emerging area of research, this study can establish an evidence base for future research using optimized interventions. For example, future research could establish the efficacy of interventions when human support is added [103,162-164], with an emphasis on when and for whom human support adds value given the effectiveness and lower cost of unguided interventions [162,166]. As recommended by Nahum-Shani et al [94], future research is required to explore how best to add *participant-initiated* or *on-demand* features to this JITAI to ensure that personal volition is balanced with planned, externally initiated support. Future iterations of *GamblingLess: In-The-Moment* could also combine EMA data with tailoring variables from passive assessments from sensors or other technologies to provide additional contextual information, lower user burden, and enhance user awareness of behavior [102,128]. Future research using cost evaluation analyses that weigh the relative costs and outcomes of *GamblingLess: In-The-Moment* with other interventions could inform health care resource allocation decisions [100], and the efficacy of this intervention delivered as a transdiagnostic intervention to address the cognitive processes underlying all addictions could improve treatment efficacy, efficiency, and cost-effectiveness [167]. Finally, there is scope for harnessing machine learning approaches to develop accurate models identifying response patterns that predict the risk of unplanned gambling in real time, ideally with respect to targeting factors contributing to the risk of gambling for each individual [128].

### Dissemination of Findings

The findings of this evaluation of *GamblingLess: In-The-Moment* will be disseminated in peer-reviewed journal articles, conference presentations, stakeholder forums, and professional development seminars. A summary of the findings of the trial will be available on the *GamblingLess* website when they become available [168].

## Appendix

Multimedia Appendix 1 GamblingLess: In-The-Moment ecological momentary assessments items.

Multimedia Appendix 2 Overview of the GamblingLess: In-The-Moment intervention options.

Multimedia Appendix 3 GamblingLess: In-The-Moment intervention eligibility.

Multimedia Appendix 4 GamblingLess: In-The-Moment intervention eligibility.

Multimedia Appendix 5 GamblingLess: In-The-Moment user-testing scores.

## Abbreviations

EMA: ecological momentary assessment
GEE: generalized estimating equations
JITAI: just-in-time adaptive intervention
mHealth: mobile health
MRT: microrandomized trial
SPGeTTI: smartphone-based problem gambling evaluation and technology testing initiative
