# Supplementary material for: A Gambling Just-In-Time Adaptive Intervention (GamblingLess: In-The-Moment): Protocol for a Microrandomized Trial
Source: JMIR Res Protoc. 2022 Aug 23;11(8):e38958. doi: 10.2196/38958 (PMC9449828; doi:10.2196/38958)
Supplement: Multimedia Appendix 1 [file resprot_v11i8e38958_app1.docx]

## Multimedia Appendix 1: *GamblingLess: In-The-Moment* EMA items

| Construct | EMA item | Response options |
| --- | --- | --- |
| Craving intensity (tailoring variable) | How strong is your urge to gamble right now? | 1. No urge 2. Mild 3. Moderate 4. Severe 5. Extreme |
| Self-efficacy (tailoring variable) | Right now, how confident are you that you would be able to resist the urge to gamble in high-risk situations (financial pressures OR unpleasant emotions OR social pressure to gamble OR testing control over your gambling OR conflict with others)? | 1. Very confident 2. Moderately confident 3. Somewhat confident 4. Slightly confident 5. Not at all confident |
|  |  |  |
|  |  |  |
|  |  |  |
|  |  |  |
|  |  |  |
| Positive outcome expectancies (tailoring variable) | Right now, how strongly do you agree that gambling has some positive outcomes (excitement OR escaping your problems OR winning money)? | 1. Not at all agree 2. Slightly agree 3. Somewhat agree 4. Moderately agree 5. Very much agree |
|  |  |  |
|  |  |  |
|  |  |  |
| Psychological distress | Right now, how distressed are you? | 1. Not at all 2. Slightly 3. Somewhat 4. Moderately 5. Very |
| Readiness to change | Right now, where does reducing your gambling fit on your list of priorities? | 1. Not a priority at all 2. Slightly important priority 3. Somewhat important priority 4. Moderately important priority 5. Very important priority |
| Subjective alcohol intoxication | Right now, how drunk do you feel? | 1. Not at all 2. Slightly 3. Somewhat 4. Moderately 5. Very |
| Impulsivity | Right now, how impatient do you feel? | 1. Not at all 2. Slightly 3. Somewhat 4. Moderately 5. Very |
| Social context | Right now, who are you with? | 1. I’m alone 2. I’m with people who gamble 3. I’m with people who don’t gamble very much or at all |
| Gambling availability (financial) | Right now, do you have enough money to gamble? | 1. Definitely not 2. Probably not 3. Possibly 4. Probably 5. Definitely |
| Gambling availability (location) | Right now, how easy would it be to get to a gambling venue (or access a gambling website if you prefer internet gambling)? | 1. Not at all easy 2. Slightly easy 3. Somewhat easy 4. Moderately easy 5. Very easy |
| Gambling episode and expenditure (*event record*) | Have you gambled since the last time you checked in? | 1. No 2. Yes |
|  | (If yes) Since you last checked in, how much in $ have you spent gambling (optional) | $________ |
