# Supplementary material for: A Gambling Just-In-Time Adaptive Intervention (GamblingLess: In-The-Moment): Protocol for a Microrandomized Trial
Source: JMIR Res Protoc. 2022 Aug 23;11(8):e38958. doi: 10.2196/38958 (PMC9449828; doi:10.2196/38958)
Supplement: Multimedia Appendix 2 [file resprot_v11i8e38958_app2.docx]

## Multimedia Appendix 2. Overview of the *GamblingLess: In-The-Moment* intervention options

| **Intervention 1: Curbing Craving** | |
| --- | --- |
| Delay and Distract  Breathe Through It  Tense and Relax  Anti-Autopilot  Urge Surfing | Fast Forward  Urge Thinking Traps  Pros and Cons  The Benefits  Ten Steps to Stay Safe |
| **Intervention 2: Tackling Triggers** | |
| ***Group 1: Financial pressures*** | ***Group 2: Unpleasant emotions*** |
| Money Check  Budget Tracker  Debate Captain  Financial Goals  Fast Forward | Defusing Difficult Thoughts  Enjoyable Activities  Living in the Moment  Tense and Relax  Pros and Cons |
| ***Group 3: Social pressure*** | ***Group 4: Testing control*** |
| The “N” word  Just Say No  Do’s and Don’ts  Planning Ahead  Safe and Dangerous Contacts | Willpower Breakdown  Debate Captain  Seemingly Irrelevant Decisions  The Slip Chain  Safe Gambling Guidelines |
| ***Group 5: Conflict with others*** |  |
| My Style  Choice Spinner  Mix ‘n Match  “I” Statements  Do’s and Don’ts |  |
| **Intervention 3: Exploring Expectancies** | |
| ***Group 1: Excitement*** | ***Group 2: Escape*** |
| Feedback  Tense and Relax  Pros and Cons  Exciting Activities  Fast Forward  Anti-Autopilot | Feedback  Tense and Relax  Pros and Cons  Enjoyable Activities  Defusing Difficult Thoughts  Living in the Moment |
| ***Group 3: Money*** |  |
| Feedback  Money Check  Budget Tracker  Debate Captain  Fast Forward  Anti-Autopilot |  |
