# Supplementary material for: A Gambling Just-In-Time Adaptive Intervention (GamblingLess: In-The-Moment): Protocol for a Microrandomized Trial
Source: JMIR Res Protoc. 2022 Aug 23;11(8):e38958. doi: 10.2196/38958 (PMC9449828; doi:10.2196/38958)
Supplement: Multimedia Appendix 3 [file resprot_v11i8e38958_app3.docx]

Multimedia Appendix 3: *GamblingLess: In-The-Moment* intervention eligibility

| Construct | EMA item cut-points for intervention eligibility | Intervention option activated | Intervention group-specific/post-intervention EMA item |
| --- | --- | --- | --- |
| Craving intensity (tailoring variable) | (1) Mild to (4) Extreme | Curbing Cravings | How strong is your urge to gamble right now? |
| Self-efficacy (tailoring variable) | (1) Moderately confident to (4) Not at all confident  Participant selects highest-risk situation and is administered specific EMA item | Tackling Triggers |  |
|  |  | (Financial pressures group) | Right now, how confident are you that you would be able to resist the urge to gamble in situations involving financial pressures? |
|  |  | (Unpleasant emotions group) | Right now, how confident are you that you would be able to resist the urge to gamble in situations involving unpleasant emotions? |
|  |  | (Social pressure group) | Right now, how confident are you that you would be able to resist the urge to gamble in situations involving social pressure to gamble? |
|  |  | (Testing control group) | Right now, how confident are you that you would be able to resist the urge to gamble in situations involving testing control over your gambling? |
|  |  | (Conflict with others group) | Right now, how confident are you that you would be able to resist the urge to gamble in situations involving conflict with others? |
| Positive outcome expectancies (tailoring variable) | (1) Slightly agree to (4) Very much agree  Participant selects highest-endorsed positive outcome expectancy and is administered specific EMA item | Exploring Expectancies |  |
|  |  | (Excitement expectancies group) | Right now, how strongly do you agree that gambling is exciting? |
|  |  | (Escape expectancies group) | Right now, how strongly do you agree that gambling helps you escape your problems? |
|  |  | (Money expectancies group) | Right now, how strongly do you agree that gambling is a way to win money? |
