# Supplementary material for: A Gambling Just-In-Time Adaptive Intervention (GamblingLess: In-The-Moment): Protocol for a Microrandomized Trial
Source: JMIR Res Protoc. 2022 Aug 23;11(8):e38958. doi: 10.2196/38958 (PMC9449828; doi:10.2196/38958)
Supplement: Multimedia Appendix 4 [file resprot_v11i8e38958_app4.docx]

## Multimedia Appendix 4. Summary of self-report measures employed in the within-group follow-up evaluation

| Construct | Measure description | Evaluation time-point | | |
| --- | --- | --- | --- | --- |
|  |  | Pre-interv-ention | Post-interv-ention | Six-month follow-up |
| **Descriptive and covariate measures** | | | | |
| Socio-demographic characteristics | Participant type (gambler, family member/friend, clinician/researcher/policy maker), age, gender, country of residence, residential postcode, ethnicity, and annual personal gross income. | X |  |  |
| Problem gambling activity | Dichotomously-scored items measuring whether participants think they have an issue with: number games like lotteries, keno, powerball or bingo; pokies or electronic gaming machines; informal private betting for money like playing cards at home; table games like blackjack, roulette, and poker; horses or harness racing or greyhounds; and sports or event results. | X |  |  |
| Intended gambling behaviour (frequency, expenditure) | Intended gambling behaviour will be measured at pre-intervention using a novel adaptation of the TimeLine Follow-Back (TLFB) [148], a 28-day TimeLine Follow-Forward (TLFF). At post-intervention, intended gambling behaviour will be measured using two single items: (1) *Over the next six months, how many days per month do you intend to gamble?* (intended gambling frequency) and (2) *Over the next six months, how much money per month do you intend to spend on gambling?* (intended gambling expenditure). | X | X |  |
| Help-seeking | The Help-Seeking Questionnaire [149] will be employed at post-treatment and follow-up to identify the frequency of accessing high-intensity and low-intensity support in the previous 28 days and 6 months, respectively. High-intensity gambling interventions (5 items) will include: talking to a gambling counsellor face-to-face; attending a gambling support group; talking to a psychologist, psychiatrist or GP about gambling; seeking financial counselling by phone or face-to-face; and staying in a residential gambling treatment facility. Low-intensity gambling interventions (3 items) will include: phoning a gambling helpline; talking to a gambling counsellor online via chat; and sending an email to a gambling counsellor. An additional self-directed help-seeking item measuring exclusion from a land-based venue or online gambling website will also be included in this scale; this item will be included as a low-intensity gambling intervention in this trial. |  | X | X |
| **Primary outcome measure** | | | | |
| Gambling symptom severity | The Gambling Symptom Assessment Scale (G-SAS) [129] is a 12-item self-report scale designed to assess changes in gambling symptom severity during treatment. Each G-SAS item employs a past-week timeframe and is scored from 0 to 4, with varying response options. Total scores on the G-SAS range from 0 to 48, with higher scores indicating greater gambling symptom severity. Scores on the G-SAS are categorised as extreme (41–48), severe (31–40), moderate (21–30) or mild (8–20). The G-SAS has demonstrated high internal consistency (α=0.87) and good convergent validity with other measures of gambling symptom severity [130]. | X | X | X |
| **Secondary outcome measures** | | | | |
| Gambling behaviour (frequency, expenditure) | Gambling frequency and expenditure will be assessed using a 28-day TLFB [148] at pre-intervention; using the 28-day *e*-EMA data at post-intervention; and using two single items at follow-up: (1) *Over the last six months, how many days per month have you gambled?* and (2) *Over the past six months, how much money per month have you spent on gambling?*. | X | X | X |
| Craving severity | The 5-item Penn Alcohol Craving Scale [X] measures overall alcohol craving severity in the week prior to the assessment, including frequency and time spent thinking about alcohol, difficulty in resisting drinking opportunities, and strength of craving episodes. Items are scored on varying 7-point scale, ranging from (0) *Never, None, Not at all* to (6) *Nearly all, Strong, All the time*, resulting in scores from 0 to 30. The gambling adaptation of this scale, the Penn Gambling Craving Scale [25], has replaced the “drinking” words with “gambling” words. | X | X | X |
| Self-efficacy | Confidence in ability to resist gambling when faced with high-risk situations will be measured using the Brief Situational Confidence Questionnaire (BSCQ) [46] adapted for gambling. The BSCQ employs a visual analogue scale from (0%) *Not at all confident* to (100%) *Totally confident*. The original BSCQ has been adapted to gambling (BSCQ-G), which measure self-efficacy across eight types of situations: unpleasant emotions, physical discomfort, pleasant emotions, financial pressures, urges and temptations, conflict with others, social pressure to gamble, and filling time [130]. Scores on the BSCQ-G can range from 0 to 800, with scores of 640+ representing high confidence, 480-639 representing moderate self-confidence, and scores of below 480 representing low self-confidence. Testing control was added to this measure, given that *GamblingLess: In-The-Moment* targets self-effiacy in this situation. The original BSCQ demonstrates excellent internal consistency (α =.85) [46]. | X | X | X |
| Positive outcome expectancies | Positive outcome expectancies will be measured using the Excitement (3 items), Escape (4 items), and Money (3 items) subscales of the Gambling Outcome Expectancies Scale (GOES) [69]. The GOES comprises five subscales assessing gambling expectancies using belief-based statements about the perceived outcomes of gambling, independent of gambling frequency. In an Australian adaptation of this scale employed in an Australian representative general population sample, each item was modified to be rated on a 5-point Likert scale from (1) *Strongly Disagree* to (5) *Strongly Agree* [76]. The subscale scores are computed by averaging the item scores. In the Australian sample, at-risk gamblers were more likely to endorse excitement, escape, and money outcome expectancies than low-risk gamblers [76]. Psychometric evaluation reveals the GOES displays good temporal stability and that the selected subscales display good internal consistency coefficients: Excitement (α =.77-.85), Escape (α =.86-.88), and Money (α =.81-.92) [69, 76, 78]. | X | X | X |
