# Supplementary material for: A Gambling Just-In-Time Adaptive Intervention (GamblingLess: In-The-Moment): Protocol for a Microrandomized Trial
Source: JMIR Res Protoc. 2022 Aug 23;11(8):e38958. doi: 10.2196/38958 (PMC9449828; doi:10.2196/38958)
Supplement: Multimedia Appendix 5 [file resprot_v11i8e38958_app5.docx]

## Multimedia Appendix 5. *GamblingLess: In-The-Moment* user testing scores

| Mobile App Rating Scale subscale scores (1-5) | | | M (SD) |
| --- | --- | --- | --- |
|  | Engagement | | 3.57 (0.70) |
|  | Functionality | | 3.62 (0.59) |
|  | Aesthetics | | 3.85 0.52) |
|  | Information | | 3.99 (0.58) |
|  | Overall App Quality | | 3.75 (0.51) |
|  | Subjective Quality | | 4.21 (1.08) |
|  | Perceived Impact | | 3.40 (0.58) |
|  |  | Awareness | 3.31 (1.11) |
|  |  | Knowledge | 3.69 (0.86) |
|  |  | Attitudes | 3.31 (1.11) |
|  |  | Intention to change | 3.38 (1.12) |
|  |  | Help-seeking | 3.38 (1.04) |
|  |  | Behaviour Change | 3.31 (1.11) |
| Tailored interventions (modules) (0-10) | | |  |
|  | Curbing Cravings | |  |
|  |  | Ease of completion | 8.85 (1.07) |
|  |  | Helpfulness | 7.46 (2.75) |
|  | Tackling Triggers | |  |
|  |  | Ease of completion | 8.00 (1.58) |
|  |  | Helpfulness | 7.23 (2.83) |
|  | Exploring Expectancies | |  |
|  |  | Ease of completion | 8.15 (1.57) |
|  |  | Helpfulness | 7.00 (2.61) |
| Onboarding (0-10) | | |  |
|  | Clarity of app description | | 8.00 (1.47) |
|  | Understanding of what was required | | 7.08 (2.43) |
|  | Ease of trial sign up | | 6.77 (2.12) |
| EMAs (0-10) | | |  |
|  | Relevance | | 7.38 (2.87) |
|  | Ease of completion | | 7.38 (2.87) |
| Clarity of activity explanations and instructions (0-10) | | | 8.69 (1.03) |
| Relevance of activities based on check-in information (0-10) | | | 7.23 (2.94) |
| Helpfulness of specific app features (0-10) | | |  |
|  | “Did You Know?” messages | | 8.46 (1.33) |
|  | “Click to Call/Email” for help services | | 8.85 (1.34) |
|  | “Pick for Me” option | | 8.62 (1.80) |
